# Diversity and evolution of rice progenitors in Australia

**DOI:** 10.1002/ece3.3989

**Published:** 2018-04-02

**Authors:** Ali M. Moner, Agnelo Furtado, Ian Chivers, Glen Fox, Darren Crayn, Robert J. Henry

**Affiliations:** ^1^ Queensland Alliance for Agriculture and Food Innovation University of Queensland Brisbane Qld Australia

**Keywords:** Australian wild rice, nuclear genes chloroplast sequence, *Oryza* AA genome, phylogenetic analysis

## Abstract

In the thousands of years of rice domestication in Asia, many useful genes have been lost from the gene pool. Wild rice is a key source of diversity for domesticated rice. Genome sequencing has suggested that the wild rice populations in northern Australia may include novel taxa, within the AA genome group of close (interfertile) wild relatives of domesticated rice that have evolved independently due to geographic separation and been isolated from the loss of diversity associated with gene flow from the large populations of domesticated rice in Asia. Australian wild rice was collected from 27 sites from Townsville to the northern tip of Cape York. Whole chloroplast genome sequences and 4,555 nuclear gene sequences (more than 8 Mbp) were used to explore genetic relationships between these populations and other wild and domesticated rices. Analysis of the chloroplast and nuclear data showed very clear evidence of distinctness from other AA genome *Oryza* species with significant divergence between Australian populations. Phylogenetic analysis suggested the Australian populations represent the earliest‐branching AA genome lineages and may be critical resources for global rice food security. Nuclear genome analysis demonstrated that the diverse *O. meridionalis* populations were sister to all other AA genome taxa while the Australian *O. rufipogon*‐like populations were associated with the clade that included domesticated rice. Populations of apparent hybrids between the taxa were also identified suggesting ongoing dynamic evolution of wild rice in Australia. These introgressions model events similar to those likely to have been involved in the domestication of rice.

## 1 INTRODUCTION

Rice (*Oryza sativa* L.) is a critically important cereal crop being a key source of carbohydrates (calories) and an important source of many other nutrients for more than half of the world's people (Civáň, Craig, Cox, & Brown, 2015; Huang et al., 2012). The wild relatives of rice represent a valuable resource for rice improvement and adaptation to meet the needs of a growing human population in a changing environment (Henry, 2016; Henry et al., 2010; Mickelbart, Hasegawa, & Bailey‐Serres, 2015).

Wild *Oryza* species are widespread in northern Australia (Henry et al., 2010). This is an area without a long history of rice cultivation, implying that the wild populations have remained largely isolated from the impacts of gene flow from domesticated crops that has apparently been widespread in Asia (Brozynska et al., 2017). The AA genome species of rice include cultivated species and their close relatives (Choi, Platts, Fuller, Wing, & Purugganan, 2017). Draft genome sequences of the AA genome populations from Australia have recently been reported indicating that these populations may be an important genetic resource for rice because of their high diversity and phylogenetic relationship to domesticated rice (Brozynska, Furtado, & Henry, 2015; Brozynska et al., 2014, 2017; Sotowa et al., 2013; Wambugu, Brozynska, Furtado, Waters, & Henry, 2015).

We now report on an analysis of the genomes of rice collected from sites over a wide area in northeastern Australia allowing analysis of the diversity and relationships within and between these wild populations.

## 2 MATERIAL AND METHODS

### 2.1 Field collections

**Figure 1:**
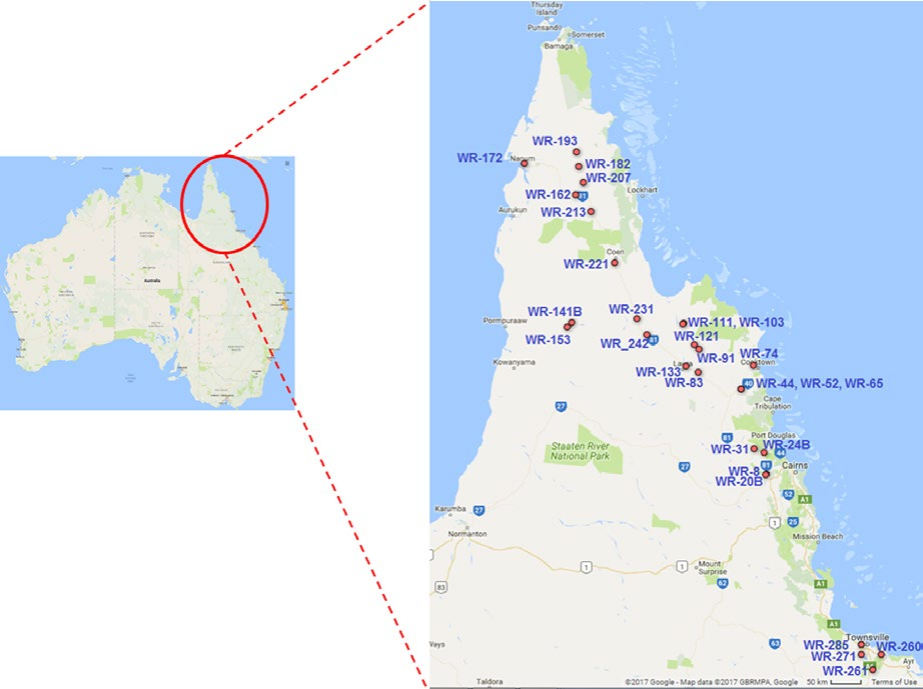
Australian wild rice collection sites. Red dots indicate collection sites

Samples and data were collected during May 2015, 2016, and 2017, from northeastern Queensland, Australia. Collections ranged from south of Townsville to the most northerly parts of Cape York Peninsula (Figure 1). Seeds and vegetative material were collected from 29 sites. GPS coordinates, observations of plant spike form, awn length, an herbarium voucher, and photographs of flowers (where possible) were obtained at each site (Appendix S1, Table S1, Figure S1).

### 2.2 Morphological measurement

Anther and awn measurements were recorded in the field. For anther length, 4–8 flowers from 3 to 6 immature panicles were selected at random from each population, photographed against a standard background with a scale, and measurements obtained later in the laboratory using Image‐Pro Plus software (Media Cybernetics, MD, USA, http://www.mediacy.com/index.aspx?page=IPP). The awn length was measured for ten different plants from each population selected at random.

### 2.3 DNA extraction and sequencing

Vegetative tissue from 29 samples (representing each of the collection sites) was prepared and DNA extracted as described by Furtado (2014). Three approaches were used to assess the quality and quantity of the extracted DNA: Nano Drop (Thermo Fisher Scientific), agarose gel electrophoresis, and Qubit (Thermo Fisher Scientific). Multiplex sequencing of the 29 wild rice samples was conducted using a Hiseq 4000 (Illumina) using 2 × 150 paired end technique, aiming to produce approximately 10× whole genome coverage on average. Reference chloroplast genome sequences were obtained as described in (Appendix S1, Table S9).

### 2.4 Chloroplast genome assembly

The sequence reads were analyzed using CLC Genomic workbench V.9, Geneious V.9.1.5 and Clone Manager Professional 9 (Kim et al., 2015). A quality check (QC) was applied to all raw data. Based on the results of the QC report, reads were trimmed. A dual pipeline approach was used to assemble the chloroplast genome sequences: mapping reads to reference and de novo assembly. The outputs of both pipelines were combined, and all discrepancies were resolved and corrected manually.

### 2.5 Chloroplast phylogenetic analysis

The assembled chloroplast genome sequences together with those that were obtained from earlier studies (a total of 42) were analyzed using Geneious V 9.1.5 (geneious.com). Chloroplast genomes were aligned using the MAFFT (MAFFT v7.308 Algorithm: auto, scoring matrix: 1PAM/k = 2 gap open penalty:1.53 offset value:0.123) plugin tool (Katoh, Misawa, Kuma, & Miyata, 2002). The alignment file was inspected physically. Bayesian inference (BI), maximum likelihood (ML), and maximum parsimony (MP) approaches, using the software packages MrBayes (Huelsenbeck & Ronquist, 2001), PHYLM (Carbonell‐Caballero et al., 2015; Guindon & Gascuel, 2003), PAUP (Swofford, 2002), respectively, were utilized to infer the evolutionary relationships. (Appendix S1, Table S6). Genetic diversity for the whole chloroplast calculated using DnaSP software (Rozas, Sánchez‐DelBarrio, Messeguer, & Rozas, 2003).

### 2.6 Chloroplast genome annotation

All chloroplast sequences were annotated using the CpGAVAS website (http://www.herbalgenomics.org/0506/cpgavas/analyzer/home), using the default parameters as recommended. The outcome was imported directly into Geneious software to allow comparison with the reference *O. sativa japonica* NC_001320 to identify polymorphisms.

### 2.7 Phylogenetic analysis of nuclear genes

Phylogenetic analysis was based upon a set of 4,643 genes that were found in all include *Oryza* species (Brozynska et al., 2017). These sequences were obtained from the sequence data pool for each field sample and reference genome using the software packages FastQC, BWA, Samtools, bcftools, and MUMmer. The accession identifiers of the reference samples used were as follows: *O. sativa japonica* AA GCA_000005425.2, *O. sativa indica* AA GCA_000004655.2, *O. rufipogon* AA GCA_000817225.1, *O. nivara* AA GCA_000576065.1, *O. barthii* AA GCA_000182155.3, *O. glaberrima* AA GCA_000147395.2, *O. glumaepatula* AA GCA_000576495.1, *O*. *meridionalis* AA GCA_000338895.2, Taxon A AA LONB00000000, Taxon B AA LONC00000000, and *O*. *punctata* BB GCA_000573905.1. A total of 4,555 genes were obtained from all samples and references. These genes were divided into groups based upon the chromosomal location in *O. sativa japonica*. Multiple sequence alignment was performed at the gene level using MAFFT (Katoh et al., 2002). Following this individual gene alignment, files were concatenated into single alignment for each chromosome; then, all chromosomes were combined into a whole genome alignment of 8,179,015 base pairs (Figure 3b).

Phylogenetic trees were reconstructed using three analytical approaches: ML, MP, and BI. For the ML analysis, PHYML version 20131022 was used with the following settings: tree topology search: NNIs, initial tree = parsimony, model of nucleotide substitution = GTR (Guindon & Gascuel, 2003). For the MP analysis, PAUP 4.0 was used with the following setting: stepwise taxon addition with random seed, heuristic tree search strategy, and 1,000 bootstrap (Swofford, 2002). For the BI analysis, MrBayes was used with same as reported in Brozynska et al. (2017).

## 3 RESULTS AND DISCUSSION

Wild AA genome rice was collected from 27 sites in north Queensland, Australia (Figure 1 and Appendix S1, Table S1). Plants were found around the margins of lakes and creeks (Appendix S1, Figure S1) where for the most part, water was available to support their growth. Wild rice was not located on Cape York north of the Jardine River (−11.103665, 142.283901) or on the Islands of Torres Strait, consistent with Herbarium records (AVH, accessed 30 June 2017). Although the cause of this distributional gap, and its temporal dynamics, is unclear, it may represent a contemporary barrier to gene flow with populations to the north in New Guinea and South East Asia.

Wild plants in the field showed significant morphological variation (Table S1), particularly in spike morphology, awn length, and anther length. Awn length varied more than threefold between sites with the open panicle types (*O. rufipogon*‐like, Taxon A) having shorter awns than the closed panicle types (*O. meridionalis‐*like, Taxon B). The shortest anthers (c. 1.5 mm) were found in plants resembling *O. meridionalis* or taxon B. In contrast, the longest anthers (4.5 mm) were found in plants resembling *O. rufipogon* or taxon A. Both awn and anther length showed highly significant (*p* < .01) differences between sites. The results agree with previous studies of these Australian populations. (Brozynska et al., 2014; Sotowa et al., 2013; Waters, Nock, Ishikawa, Rice, & Henry, 2012).

All regions of the chloroplasts were successfully sequenced. The high sequence coverage ensured a complete genome sequence was obtained for all sites in the assembly pipeline that was used. The average coverage of the total chloroplast for all samples was 683× while the highest and lowest coverages were 2,063× and 10×, respectively (Appendix S1, Table S2). Compared to the reference sequence, an average of 129.6 variants (deletions, insertions, and SNPs) per sample were found (Appendix S1, Table S3), which agrees with the results reported by Brozynska et al. (2014). A total of 18 functional polymorphisms were found in the chloroplasts with six of them common to all samples (Appendix S1, Tables S4, S5).

**Figure 2:**
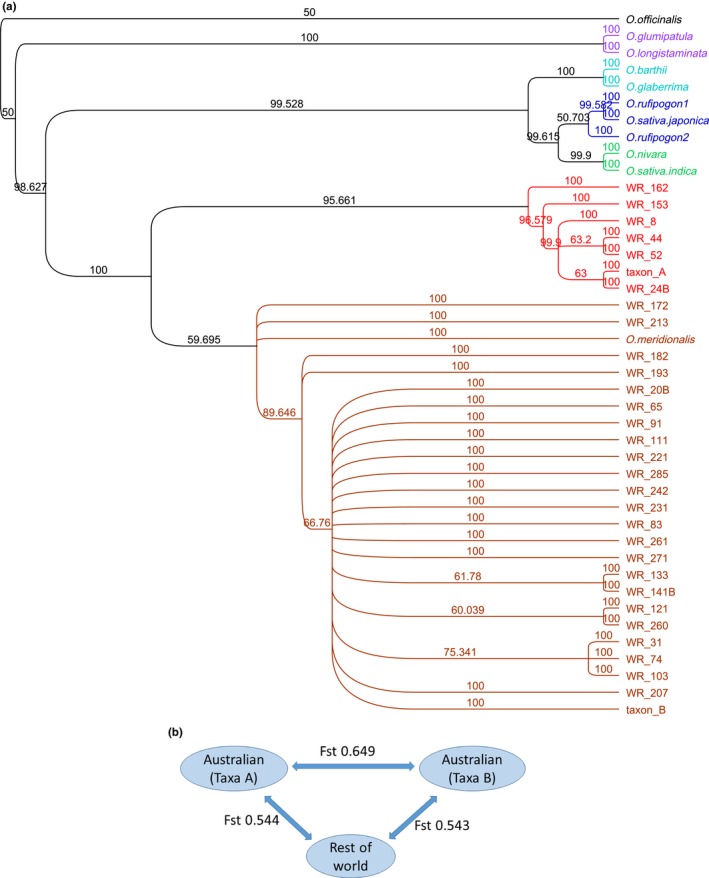
Diversity of chloroplast genomes. (a) Phylogenetic tree based on MP analysis of whole chloroplast genome sequences. Colors relate to the main clades. Red and brown clades are from Australia. Bootstrap values (MP 1,000 replicates) are shown on the branches; (b) genetic distances between populations in Australia and elsewhere

**Figure 3:**
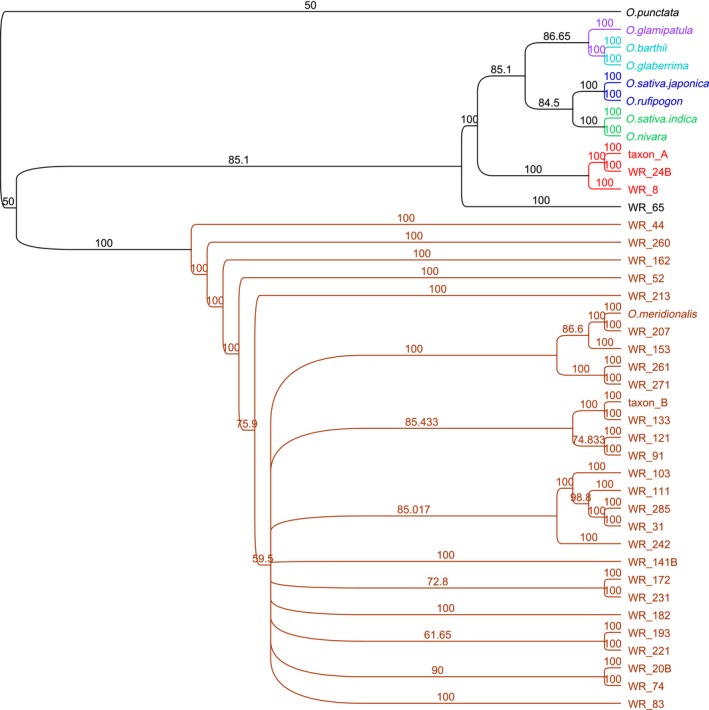
Diversity of nuclear genomes. (a) Phylogenetic tree based on MP analysis of the concatenated alignment of all nuclear genes. Colors relate to the main clades. Red and brown clades are from Australia. Bootstrap values (maximum parsimony, 1,000 replicates) are shown on the branches; (b) individual chromosome analysis showing

The aligned sequence comprised 135,532 bp. Of the variable sites, 227 were parsimony‐informative and 661 were uninformative (427 were unique). The phylogenetic trees constructed using different approaches (Appendix S1, Table S6) were highly congruent (Brozynska et al., 2014; Kim et al., 2015; Wambugu et al., 2015). As in earlier work (Wambugu et al., 2015), a clade including *O. glumipatula* and *O. longistaminata* was sister to all other AA genome rices which were divided into an Australian clade, and a clade with Asian and African taxa including the two domesticated species. The Australian clade contained two main clades: a small clade (7 populations) containing Taxon A and a much larger clade (20 populations) containing the majority of the samples including Taxon B and *O. meridionalis*. This result confirms that the chloroplast genome of Taxon A is not closely related to that of Asian *O. rufipogon* despite the plants having a similar appearance. Eight unique chloroplast molecular makers were found in all members of the clade that includes Taxon A (Appendix S1, Table S7) (Kim et al., 2015). The chloroplasts of the different Australian AA genome taxa showed significant genetic differences (Figure 2). The concatenated alignment of 4,555 nuclear genes comprised 8,179,015 bp of which 44.1% were invariant. The minimum and maximum lengths were 5,916,081 and 7,013,653 bp, respectively, slightly longer than reported previously (Brozynska et al., 2017). The nuclear analysis (as one full length sequence and by chromosomes) grouped the Australian samples into two main clades. One of these included Taxon A and the other much larger group (27 samples) included Taxon B and *O*. *meridionalis* types (Figure 3). This analysis confirmed the nuclear genomes of the diverse *O. meridionalis* group including Taxon B are sister to those of all other AA genome taxa. However, four other Australian samples including Taxon A grouped within the clade that includes all other AA genome species as suggested by the single genome analysis of (Brozynska, et al. 2017). The phylogeny based upon individual chromosomes (Appendix S1, Figure [Link], [Link], [Link], [Link], [Link], [Link], [Link], [Link], [Link], [Link], [Link], [Link]) shows that these populations were a sister to all Asian and African rices (chromosomes 4, 5, 6, 7, 8) or the Asian rices (chromosome 9, 10), *O. indica/O. nivara* (1, 2, 3, 11) or Australian (12) clades indicating significant introgression between the different populations of wild rice.

The chloroplast genomes of Taxon B are diverse and include a small number (populations WR‐44, WR‐52. WR‐153, WR‐162) that showed close relationships to the chloroplast genome found in the plants with an A genome. These included the most divergent B types (e.g., WR‐44, WR‐52 and WR‐162). Some of these were from sites where morphological traits were somewhat intermediate between the Taxon A and Taxon B types. For example, the populations found on the Lakeland‐Cooktown road had large anthers and panicles that varied from open to close. The divergent B nuclear genome and A chloroplast genome suggests plants in these populations may be hybrids. Population WR‐65 had a B‐type chloroplast but an A‐type nuclear genome.

Both chloroplast and nuclear gene analysis suggest a high diversity of AA genome wild rice in Australia. This supports the view that Australia might be a center of diversity for the AA genome clade. The populations with a morphology similar to *O. meridionalis* are diverse and may include both annual and perennial types (Brozynska et al., 2014; Sotowa et al., 2013). These populations could all be considered part of one diverse species, *O. meridionalis*. The nuclear genome analysis of the *O. rufipogon*‐like (Taxon A) populations places them in the Asian clade together with domesticated rices. This suggests these Australian populations should be considered as a distinct, undescribed taxon (Brozynska et al., 2017). Analysis of the chloroplast genomes placed Australian plants with *O. rufipogon*‐like morphology in the Australian clade, distant from the Asian *O. rufipogon* which were placed in the Asian clade. Some populations with a nuclear genome similar to *O. meridionalis* had a chloroplast genome that was closer to the *O. rufipogon*‐like plants (Taxon A) suggesting that their evolutionary history involved some introgression or hybridization and chloroplast capture (Brozynska et al., 2014, 2017; Wambugu et al., 2015). One example of chloroplast capture in the other direction was also detected (WR‐65). This illustrates a dynamic state of evolution of wild *Oryza* in Australia. This type of ongoing introgression is demonstrated by the analysis of the individual chromosomes in these populations and similar events may explain the domestication of wild indica by introgression of domestication alleles from domesticated japonica (Civan 2015). Extensive evidence shows distinct wild progenitors populations for indica and japonica rice that require separate domestication (Civan 2015) while the presence of common domestication related alleles suggests a single domestication event (Huang 2012). The discovery of natural hybrids between taxa with greater divergence than indica and japonica demonstrates the potential for similar hybridization events to be associated with the transfer of domestication‐related alleles during rice domestication.

Further research should determine the diversity of useful alleles in these populations that might be incorporated into domesticated rice to improved stress tolerance and grain quality. The need for increased efforts to conserve these species in situ and ex situ is suggested by the very limited collection of this material in seed collections and the more limited distribution of the *O. rufipogon‐*like populations in the wild in locations that may be threatened by the incursion of weeds.

## CONFLICT OF INTERESTS

The authors declare no competing financial interests.

## AUTHOR CONTRIBUTIONS

R.H. conceived the project. I.C., D.C. G.F., A.M.M, A.F., and R.H. collected field samples. A.M.M., A.F., and R.H. analyzed the data and wrote manuscript. All authors read and edited the manuscript.
