# Supplementary figures and images for: Diversity and evolution of rice progenitors in Australia
Source: Ecol Evol. 2018 Apr 2;8(8):4360–6. doi: 10.1002/ece3.3989 (PMC5916314; doi:10.1002/ece3.3989)

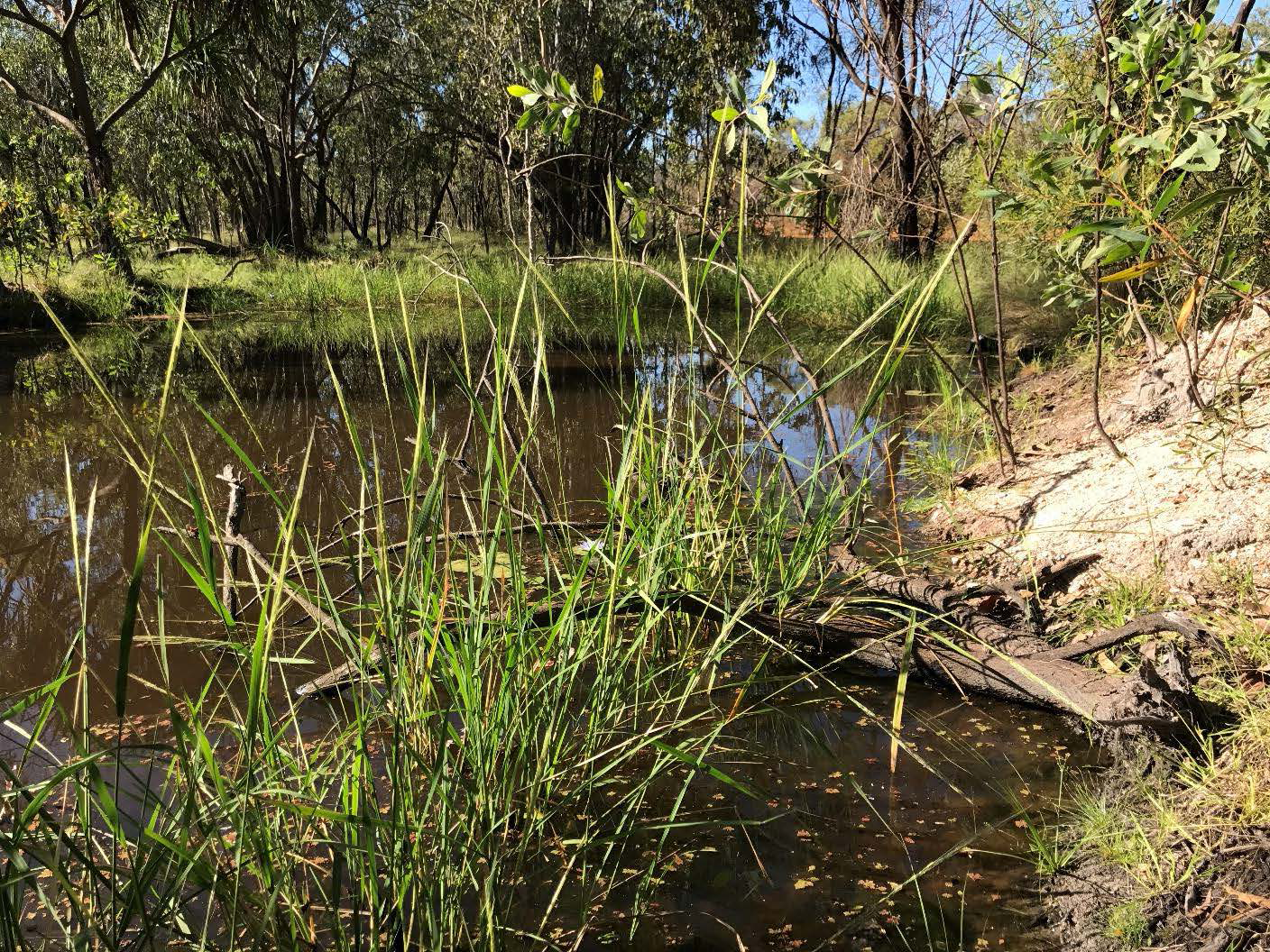

Supplement: Supplementary file 1 [file ECE3-8-4360-s001.tif]
