# Supplementary material for: Diversity and evolution of rice progenitors in Australia
Source: Ecol Evol. 2018 Apr 2;8(8):4360–6. doi: 10.1002/ece3.3989 (PMC5916314; doi:10.1002/ece3.3989)
Supplement: Supplementary file 14 [file ECE3-8-4360-s014.docx]

**Appendix 1 Supplementary information**

**Diversity and Evolution of Rice Progenitors in Australia**

Table S1. Details of collections of wild rice from north Queensland made in 2015, 2016 and 2017. Including site description, GPS coordinates,

panicle shape, awn and anther length for wild populations from each collection site.

| Site number | Sample number | Site description | GPS location and elevation | Likely Species* | Panicles | Awn  length**  (mm) | Awn SD± | Anther length  (mm)*** | Anthers SD ± |
| --- | --- | --- | --- | --- | --- | --- | --- | --- | --- |
| 1 | WR-8 | Mareeba Wetlands (Clancy Lagoon) | S:16.92661° E:145.35620° Elevation: 410 m | Taxon A | Open | 4.6 | 1.3 | 4.60 | 0.21 |
| 2 | WR-20B | Mareeba Wetlands (Pandanus lake) | S:16.93795° E:145.35077° Elevation: 422 m | Taxon B | Closed | 9.5 | 1.3 | 2.09 | 0.13 |
| 3 | WR-24B | Abbatoir Swamp (Mossman-Mt Molloy Road) | S:16.63574° E:145.32603° Elevation: 422 m | Taxon A | Open | 5 | 2.1 | - | - |
| 4 | WR-31 | small roadside swamp, cnr Bethel Road and Mulligan Hwy | S:16.57874° E:145.18906° Elevation: 363 m | Taxon B ( classic) or *O*. *meridionals* | Closed | 10 | 1.8 | 2.28 | 0.07 |
| 5 | WR-44,  WR-52 and WR-65 | Lakeland-Cook Town section, Mulligan Hwy | S:15.758640° E:144.99924° Elevation: 159 m | Mixed Taxon A and B | Open | 6.9 | 2.1 | 4.45  3.82  3.5 | 0.18 (2015 collection)  0.10 (2017collection) large lake  0.17 (2017collection) small lake |
|  |  |  |  | Taxon B | Closed | 9 | 1.3 | 4.26  2.81 | 0.23 (2017collection) large lake  0.14 (2017collection) small lake |
|  |  |  |  | Taxon B+ *O. australiensis* | Partially open | 11.8 | 1.1 |  | - |
| 6 | WR-74 | Barretts Road, near Cook Town Airport. Wetland/Swamp | S:15.43399° E:145.17816° Elevation: 25 m | *O. meridionalis*, Taxon B, Taxon B+ | Closed | 10.1 | 2.2 | 2.34 | 0.21 |
| 7 | WR-83 | Unnamed marshland/wetland | S:15.53078° E:144.38336° Elevation: 95 m | *O. meridionalis* | Closed | 8 | 2.1 | 2.08 | 0.16 |
| 8 | WR-91 | Lakefield National Park | S:15.20969° E:144.38966° Elevation: 58 m | *O. meridionalis*, Taxon B, Taxon B+ | Closed | 11.6 | 2.6 |  | - |
| 9 | WR-103 | Lakefield National Park | S:14.85996° E:144.16586° Elevation: 32 m | *O. meridionalis,* Taxon B, Taxon B+ | Closed | 8.9 | 1.3 |  | - |
| 10 | WR-111 | Jpn11 site (Sotowa, et al. 2013) | S:14.84947° E:144.16811° Elevation: 21 m | *O. meridionalis*, Taxon B, Taxon B+ | Closed | 9.1 | 0.9 |  | - |
| 11 | WR-121 | Lakefield National Park | S;15.14672° E:144.32773° Elevation: 57 m | *O. meridionalis* , Taxon B, Taxon B+ | Closed | 7.8 | 1.1 |  | - |
| 12 | WR-133 | Jpn2 site (Sotowa, et al. 2013) | S:15.43943° E:144.21111° Elevation: 148 m | *O. meridionalis,* Taxon B, Taxon B+ | Closed | 7.5 | 1.6 | 2.05 | 0.09 |
| 13 | WR-141B | Balurga Road (off Musgrave to Pormpurraw road) | S:14.83915° E:142.56808° Elevation: 88 m | Taxon B*, O. meridionalis* | Closed | 14.7 | 2.2 | 1.94 | 0.21 |
| 14 | WR-153 | Balurga Road (off Musgrave to Pormpurraw road) | S:14.90241° E:142.49919° Elevation: 75 m | Taxon B, *O. meridionalis* | Closed | 9.5 | 1.7 | 2.41 | 0.08 |
| 15 | WR-162 | Merluna | S: 13.05811° E:142.61964° Elevation: 137 m | *O. meridionalis* , Taxon B, Taxon B+ | Closed | 8.1 | 1.7 |  | - |
| 16 | WR-172 | Andoom Road, Weipa | S:12.61513° E:141.89191° Elevation : 8 m | *O. meridionalis*, Taxon B, Taxon B+ | Closed | 9.9 | 1.5 | 2.21 | 0.19 |
| 17 | WR-182 | Lydia Creek, Batavia Downs Road | S:12.66010° E:142.66843° Elevation: 68 | Taxon B, *O. meridionalis* | Closed | 10.9 | 2.6 | 2.26 | 0.18 |
| 18 | WR-193 | Development road to Bamaga, Moreton. | S:12.45885° E:142.63562° Elevation: 39 | Taxon, *O. meridionalis* | Closed | 9.7 | 1.9 | 2.68 | 0.14 |
| 19 | WR-207 | Telegraph Road (Weipa turnoff to Batavia Downs). | S:12.88274° E:142.73929° Elevation: 93 | *O. meridionalis* Taxon B, Taxon B+ | Closed | 7.4 | 1.7 | 1.66 | 0.15 |
| 20 | WR-213 | Peninsular Development Road (Between Archer River Road to Weipa turnoff) | S:13.29167° E:142.84729° Elevation: 148 | *O. meridionalis*, Taxon B, Taxon B+ | Closed | 11.2 | 1.6 | 1.54 | 1.03 |
| 21 | WR-221 | Peninsula Development Road (Between Coen and Musgrave) | S:14.005117° E:143.1903607° Elevation: 208 | *O. meridionalis*, Taxon B, Taxon B+ | Closed | 8.6 | 1.7 | 2.19 | 0.18 |
| 22 | WR-231 | Peninsula Development Road (Between Musgrave to Laura) | S:14.785617° E:143.504467° Elevation: 76 | *O. meridionalis*, Taxon A+ or *O*. *officinalis* | Open /completely open | 9 | 1.7 | 2.51 | 0.11 |
| 23 | WR-242 | Peninsula Development Road | S:15.00745° E:143.640993° Elevation: 59 | Taxon, *O. meridionalis* | Closed | 6.6 | 1.1 | 1.69 | 0.14 |
| 24 | WR-260 | Townsville Site-1, Bruce Highway 30 km south of Townsville | S:19.395962  E:147.004486 | Taxon B | Closed | 9.3 | 1.4 | 2.03 | 0.12 |
| 25 | WR-261 | Townsville Site-2, Woodstock-Giru Road | S:19.599657  E:146.882965 | Taxon A, ? | Open / Closed | 5.9 | 1.3 | 1.83 | 0.07 |
| 26 | WR-271 | Townsville Site-3, Charters Towers-Townsville road | S:19.397224  E:146.723831 | Taxon A, ? | Open / Closed | 6.0 | 2.7 | 1.90 | 0.08 |
| 27 | WR-285 | Townsville Site-4,  Town Common Wetlands, Townsville | S:19.25445  E:146.725586 | Taxon B, *O. meridionalis* | Closed | 10.5 | 1.7 | 3.60 | 0.17 |

*Designation in field: Taxon A *Oryza rufipogon*-like (open panicles), Taxon B *O. meridionalis* (closed panicles and short anthers) and Taxon B+ different to both Taxon A and B.

**Awn length average in cm for 10 seeds from 10 different plants from the population sampled randomly. Not representing the sequenced sample

***this is the average of ten anthers from the same plant. Not representing the sequenced sample

±standard deviation

‡ this site contains three different taxa

Table S2. Details of sequence coverage of Australian wild rice samples. Including whole genome coverage with total number of reads, and minimum, maximum and mean coverage of the chloroplast genome.

|  | Sample number | Site number | Whole genome | | Chloroplast genome | | |
| --- | --- | --- | --- | --- | --- | --- | --- |
|  |  |  | Sequencing coverage | Total reads | Minimum coverage | Maximum coverage | Mean coverage |
| 1 | WR-8 | 1 | 7.33 | 16,581,166 | 10 | 649 | 388.07 |
| 2 | WR-20B | 2 | 9.21 | 20,821,128 | 16 | 620 | 364.43 |
| 3 | WR-24B | 3 | 10.2 | 23,069,168 | 24 | 1008 | 646.98 |
| 4 | WR-31 | 4 | 9.35 | 21,140,048 | 17 | 659 | 446.06 |
| 5 | WR-44 | 5 | 8.11 | 18,332,596 | 17 | 503 | 310.81 |
| 6 | WR-52 | 5 | 8.61 | 19,462,696 | 15 | 610 | 370.77 |
| 7 | WR-65 | 5 | 8.79 | 19,873,876 | 23 | 1082 | 718.52 |
| 8 | WR-74 | 6 | 9.84 | 22,243,622 | 21 | 863 | 579.19 |
| 9 | WR-83 | 7 | 15.42 | 34,862,816 | 34 | 1054 | 685.87 |
| 10 | WR-91 | 8 | 13.3 | 30,070,336 | 35 | 1337 | 922.73 |
| 11 | WR-103 | 9 | 12.24 | 27,683,838 | 47 | 1507 | 1088.64 |
| 12 | WR-111 | 10 | 13.62 | 30,802,742 | 41 | 1314 | 961.82 |
| 13 | WR-121 | 11 | 11.22 | 25,377,232 | 35 | 1011 | 686.18 |
| 14 | WR-133 | 12 | 7.74 | 17,509,322 | 19 | 608 | 408 |
| 15 | WR-141B | 13 | 14.48 | 32,739,902 | 24 | 1128 | 700.56 |
| 16 | WR-153 | 14 | 10.88 | 24,591,888 | 33 | 1330 | 906.44 |
| 17 | WR-162 | 15 | 5.63 | 12,732,082 | 20 | 587 | 400.76 |
| 18 | WR-172 | 16 | 6.9 | 15,604,400 | 12 | 476 | 278.41 |
| 19 | WR-182 | 17 | 8.42 | 19,030,168 | 47 | 1268 | 901.65 |
| 20 | WR-193 | 18 | 13.22 | 29,898,648 | 46 | 1541 | 1104 |
| 21 | WR-207 | 19 | 8.71 | 19,686,052 | 21 | 821 | 577.54 |
| 22 | WR-213 | 20 | 8.42 | 19,029,062 | 33 | 914 | 575.42 |
| 23 | WR-221 | 21 | 10.37 | 23,450,552 | 56 | 1283 | 930.66 |
| 24 | WR-231 | 22 | 6.29 | 14,225,150 | 22 | 646 | 412.28 |
| 25 | WR-242 | 23 | 11.42 | 25,826,240 | 56 | 2063 | 1444.48 |
| 26 | WR-260 | 24 | 3.95 | 8,934,498 | 15 | 478 | 320.6 |
| 27 | WR-261 | 25 | 11.47 | 25,936,014 | 50 | 1341 | 932.98 |
| 28 | WR-271 | 26 | 13.89 | 31,419,206 | 58 | 1518 | 1015.09 |
| 29 | WR-285 | 27 | 9.55 | 21,591,668 | 48 | 1085 | 738.28 |

Table S3. Variants in chloroplast genomes insertions, deletions and SNPs compared with the *O. sativa* subsp. *japonica* Nipponbare GU592207.1 reference genome. Abbreviations are as follows: Del: deletion, Del.T.R.: deletion tandem repeat, Ins.: insertion, Ins.T.R.: insertion tandem repeat, SNP Tr.: SNP transition, SNP Trv.:SNP transversion and Subs.: substitution.

| Sample number | Deletion | Deletion tandem repeat | Insertion | Insertion tandem repeat | SNP transetion | SNP transversion | Substitution | Total |
| --- | --- | --- | --- | --- | --- | --- | --- | --- |
| WR-8 | 12 | 7 | 5 | 11 | 48 | 41 | 4 | 128 |
| WR-20B | 11 | 8 | 6 | 10 | 49 | 43 | 3 | 130 |
| WR-24B | 12 | 7 | 4 | 11 | 47 | 42 | 4 | 127 |
| WR-31 | 11 | 7 | 6 | 11 | 50 | 44 | 4 | 133 |
| WR-44 | 12 | 7 | 4 | 12 | 48 | 41 | 4 | 128 |
| WR-52 | 12 | 7 | 4 | 12 | 48 | 41 | 4 | 128 |
| WR-65 | 12 | 8 | 6 | 10 | 49 | 42 | 3 | 130 |
| WR-74 | 12 | 7 | 6 | 10 | 50 | 43 | 4 | 132 |
| WR-83 | 12 | 7 | 6 | 11 | 49 | 43 | 3 | 131 |
| WR-91 | 12 | 8 | 6 | 10 | 49 | 43 | 2 | 130 |
| WR-103 | 12 | 7 | 6 | 10 | 50 | 43 | 4 | 132 |
| WR-111 | 12 | 7 | 6 | 10 | 49 | 42 | 3 | 129 |
| WR-121 | 12 | 7 | 6 | 10 | 49 | 43 | 3 | 130 |
| WR-133 | 11 | 8 | 6 | 11 | 49 | 43 | 3 | 131 |
| WR-141B | 13 | 7 | 6 | 10 | 50 | 43 | 3 | 132 |
| WR-153 | 12 | 7 | 4 | 11 | 46 | 39 | 3 | 122 |
| WR-162 | 13 | 8 | 5 | 11 | 48 | 39 | 5 | 129 |
| WR-172 | 11 | 7 | 6 | 11 | 49 | 40 | 4 | 128 |
| WR-182 | 12 | 7 | 6 | 10 | 49 | 42 | 4 | 130 |
| WR-193 | 12 | 7 | 6 | 10 | 49 | 42 | 4 | 130 |
| WR-207 | 13 | 7 | 6 | 12 | 49 | 42 | 2 | 131 |
| WR-213 | 12 | 6 | 6 | 10 | 44 | 40 | 5 | 123 |
| WR-221 | 12 | 7 | 6 | 10 | 49 | 42 | 3 | 129 |
| WR-231 | 12 | 7 | 6 | 10 | 49 | 42 | 4 | 130 |
| WR-242 | 12 | 7 | 6 | 10 | 50 | 43 | 3 | 131 |
| WR-260 | 12 | 7 | 6 | 10 | 49 | 43 | 4 | 131 |
| WR-261 | 12 | 7 | 6 | 11 | 49 | 43 | 4 | 132 |
| WR-271 | 12 | 7 | 6 | 11 | 49 | 43 | 4 | 132 |
| WR-285 | 12 | 7 | 6 | 10 | 49 | 42 | 3 | 129 |

Table S4. Chloroplast functional nucleotide polymorphisms (FNPs) in Australian wild rice populations. including position, gene name, gene product, amino acid substitution and codon change.

|  | Site | Gene | Gene product | Protein ID | Amino acid change | CDS | CDS codon number | CDS position | CDS position within codon | Change | Codon change | Polymorphism type | Protein effect |
| --- | --- | --- | --- | --- | --- | --- | --- | --- | --- | --- | --- | --- | --- |
| 1 | 8,593 |  | hypothetical protein | NP_039365.1 | G -> E | hypothetical protein CDS | 82 | 245 | 2 | G -> A | GGA -> GAA | SNP (transition) | Substitution |
| 2 | 8,599 |  | hypothetical protein | NP_039365.1 | G -> E | hypothetical protein CDS | 84 | 251 | 2 | G -> A | GGG -> GAG | SNP (transition) | Substitution |
| 3 | 8,622 |  | hypothetical protein | NP_039365.1 | S -> P | hypothetical protein CDS | 92 | 274 | 1 | T -> C | TCC -> CCC | SNP (transition) | Substitution |
| 4 | 24,178 | rpoC1 | RNA polymerase beta' subunit | NP_039374.1 | N -> S | rpoC1 CDS | 567 | 1,700 | 2 | A -> G | AAT -> AGT | SNP (transition) | Substitution |
| 5 | 24,756 | rpoC2 | RNA polymerase beta'' subunit | NP_039375.1 | Q -> H | rpoC2 CDS | 10 | 30 | 3 | G -> T | CAG -> CAT | SNP (transversion) | Substitution |
| 6 | 25,897 | rpoC2 | RNA polymerase beta'' subunit | NP_039375.1 | H -> D | rpoC2 CDS | 391 | 1,171 | 1 | C -> G | CAT -> GAT | SNP (transversion) | Substitution |
| 7 | 27,695 | rpoC2 | RNA polymerase beta'' subunit | NP_039375.1 | G -> D | rpoC2 CDS | 990 | 2,969 | 2 | G -> A | GGT -> GAT | SNP (transition) | Substitution |
| 8 | 28,019 | rpoC2 | RNA polymerase beta'' subunit | NP_039375.1 | W -> L | rpoC2 CDS | 1,098 | 3,293 | 2 | G -> T | TGG -> TTG | SNP (transversion) | Substitution |
| 9 | 29,113 | rpoC2 | RNA polymerase beta'' subunit | NP_039375.1 | N -> D | rpoC2 CDS | 1,463 | 4,387 | 1 | A -> G | AAC -> GAC | SNP (transition) | Substitution |
| 10 | 29,138 | rpoC2 | RNA polymerase beta'' subunit | NP_039375.1 | Q -> P | rpoC2 CDS | 1,471 | 4,412 | 2 | A -> C | CAA -> CCA | SNP (transversion) | Substitution |
| 11 | 30,699 | atpI | ATP synthase CF0 A subunit | NP_039377.1 | D -> N | atpI CDS | 67 | 199 | 1 | G -> A | GAT -> AAT | SNP (transition) | Substitution |
| 12 | 40,251 | psaA | photosystem I P700 chlorophyll a apoprotein A1 | NP_039383.1 | R -> G | psaA CDS | 334 | 1,000 | 2 | G -> C | CGC -> CCC | SNP (transversion) | Substitution |
| 13 | 56,665 |  | acetyl-CoA carboxylase beta subunit | NP_039394.1 | S -> Y | acetyl-CoA carboxylase beta subunit CDS | 38 | 113 | 2 | C -> A | TCT -> TAT | SNP (transversion) | Substitution |
| 14 | 66,104 | rps18 | ribosomal protein S18 | NP_039408.1 | T -> N | rps18 CDS | 155 | 464 | 2 | C -> A | ACC -> AAC | SNP (transversion) | Substitution |
| 15 | 70,278 | psbB | photosystem II 47 kDa protein | NP_039411.1 | A -> T | psbB CDS | 494 | 1,480 | 1 | G -> A | GCA -> ACA | SNP (transition) | Substitution |
| 16 | 70,281 | psbB | photosystem II 47 kDa protein | NP_039411.1 | I -> F | psbB CDS | 495 | 1,483 | 1 | A -> T | ATC -> TTC | SNP (transversion) | Substitution |
| 17 | 105,906 | ccsA | cytochrome c biogenesis protein | NP_039443.1 | Y -> S | ccsA CDS | 224 | 671 | 2 | A -> C | TAT -> TCT | SNP (transversion) | Substitution |
| 18 | 124,775 |  | hypothetical protein | NP_039456.1 | M -> L | hypothetical protein CDS | 34 | 100 | 1 | A -> C | ATG -> CTG | SNP (transversion) | Substitution |

Table S5 Comparison of the SNPs, FNPs and the unique FNPs in Australian wild rice populations.

| Accession | SNP | FNP | FNPs ratio % | Common FNPs | Unique FNPs | Unique FNPs ratio |
| --- | --- | --- | --- | --- | --- | --- |
| WR-8 | 93 | 11 | 11.83 | 6 | 5 | 46 |
| WR-20B | 95 | 12 | 12.63 | 6 | 6 | 50 |
| WR-24B | 93 | 11 | 11.83 | 6 | 5 | 46 |
| WR-31 | 98 | 12 | 12.24 | 6 | 6 | 50 |
| WR-44 | 93 | 11 | 11.83 | 6 | 5 | 46 |
| WR-52 | 93 | 11 | 11.83 | 6 | 5 | 46 |
| WR-65 | 94 | 12 | 12.77 | 6 | 6 | 50 |
| WR-74 | 97 | 12 | 12.37 | 6 | 6 | 50 |
| WR-83 | 95 | 12 | 12.63 | 6 | 6 | 50 |
| WR-91 | 94 | 12 | 12.77 | 6 | 6 | 50 |
| WR-103 | 97 | 12 | 12.37 | 6 | 6 | 50 |
| WR-111 | 94 | 12 | 12.77 | 6 | 6 | 50 |
| WR-121 | 95 | 12 | 12.63 | 6 | 6 | 50 |
| WR-133 | 95 | 12 | 12.63 | 6 | 6 | 50 |
| WR-141B | 96 | 12 | 12.5 | 6 | 6 | 50 |
| WR-153 | 88 | 10 | 11.36 | 6 | 4 | 40 |
| WR-162 | 92 | 10 | 10.87 | 6 | 4 | 40 |
| WR-172 | 93 | 14 | 15.05 | 6 | 8 | 57 |
| WR-182 | 95 | 12 | 12.63 | 6 | 6 | 50 |
| WR-193 | 95 | 12 | 12.63 | 6 | 6 | 50 |
| WR-207 | 93 | 12 | 12.9 | 6 | 6 | 50 |
| WR-213 | 89 | 10 | 11.24 | 6 | 4 | 40 |
| WR-221 | 94 | 12 | 12.77 | 6 | 6 | 50 |
| WR-231 | 95 | 12 | 12.63 | 6 | 6 | 50 |
| WR-242 | 96 | 13 | 13.54 | 6 | 7 | 54 |
| WR-260 | 96 | 12 | 12.5 | 6 | 6 | 50 |
| WR-261 | 96 | 12 | 12.5 | 6 | 6 | 50 |
| WR-271 | 96 | 12 | 12.5 | 6 | 6 | 50 |
| WR-285 | 94 | 12 | 12.77 | 6 | 6 | 50 |

Table S6. Phylogenetic analysis tools applied to chloroplast genome analysis

We compared methods and found that GTR was the best method for comparing diverse Oryza genomes (Brozynska, et al., 2014; Brozynska et al. 2017) giving results consistent with known relationships at different genetic distances.

|  | Program | Analysing method | Substitution model | Rate variation | Bootstrapping | Out group |
| --- | --- | --- | --- | --- | --- | --- |
| 1 | PAUP | Maximum Parsimony | GTR | Gamma | 1000 | *O. officinalis* |
| 3 | PHYLM | Maximum likelihood | GTR | Gamma | 1000 | - |
| 4 | MrBayes | Bayesian | GTR | Gamma | 2000 | *O. officinalis* |

Table S7 Unique chloroplast SNPs found in the Australian taxa.

|  | Sequence | SNPs |
| --- | --- | --- |
| 1 | CACTAATAGGTTTCATGTTACGTCAATTTGAACTTGCTCGGTCTGTTCAATTGCG A/G CCTTATAATGCAATTTCATTCTCTGGCCCAATCGCTGTTTTTGTTTCCGTATTCCTGATTT | A Australian new taxa clade |
| 2 | GTCTTTCTGGTAGCTATTCTAAATTCTCTCATTTCTTAAATGTGTTTAGTA G/T TTAGTAGCCCG C/A TACAAAATAAAAAAGGGCCGTTTATTCGGATTGTGAGACGCATTAAAATGCAATTTGCG | G,C Australian new taxa clade |
| 3 | GCGAAGCAGGGGGGTGTAAATTGCAAAAAAGAAATTGGACTCTTTTTCCTATTAGATCA C/A TCAAATCACTACCCGTACTGAACTAATATAGAATCCCTTTTATTAATCTATTCTTATTCCATATCCTTT | C Australian new taxa clade |
| 4 | GTATTAACGATTGGAAACCGTCGAGGTATTTGTGCAAATAGATATAATAGTTGCGGAAACTATCCAAACCAAAAAGTAAG/ATTACAATAATAATAATCCTAAGTATACGAAAGATAAAGAATCTCTTTTTTCTAGTTCCTATGATGCA CTGGGAGCTTATAGACAGAAACAAAT | G Australian new taxa clade |
| 5 | CCCGCAACCCCACGGTTATGAGCCTTGTCAGCTACCAAACTGTTCTATCCTGTTAAACTAAAGAGAGGGGAACTAGTGGATAAAA A/G GGGGGTTGAATACGCCCCTCTACCATATCTATACAAATAGAATAGTC CATTTATACAGAAT GGTAAAGAGGGCTCTTCTACGATCATCAATTCCAGAAATCCAT | A Australian new taxa clade |
| 6 | AAGATTTCTCAATTTTCATTAAATCTTATAGAAAGAGGTAGAATTTCTTCTTTTTTTCAGGGATTTTAGGG AAA C/A TAAGGCTCTTGTCATTTTTTATTCTATTACTGAACAG AATGGGAAGACAGGGTTGGTTATTCTT CGTCTACGAATATCCAAATTTTAAC | C Australian new taxa clade |
| 7 | TTCGTAAAAATCTTTGGAAGAAAAAGACTTATTTTTCCATAGTACAATCTTATTCTTTAGCAAAATCAAGATCATTTTCTGGCGTCAGCGAGCA C/T CCAAAACCAAAGGGTTTTTCTCGGCAACAAACAAA CAAATAA TAGGGTTTTGGGATAATATGAATTGACCTATCCCCAAAAAATTCCAATTATTTAATATGAATAATTAG | C Australian new taxa clade |
| 8 | TCTTTTTGCCATTGGACTTTCCAATCGAATTGATTGTAAGACTCGTAAAGATCAACTTTACGAAGATCCCATTGTATTCCAGAAGCTCGTAACATGGG A/G CCCGATAAGCCCCAATTTACAGCTTCTTCTCCGCTAATAAA ACCAACTCCCTCAACTCGTTCCAAAAAAATGGGATTCTGTGTAATAAGTTGTTGATATTCAA | A Australian new taxa clade |

Table S8. Chromosomes phylogenetic analysis topology agreement.

| Chromosome | Maximum Likelihood (ML) Vs Maximum Parsimony (MP) | Maximum Likelihood (ML)Vs Bayesian Inference (BI) | Maximum Parsimony (MP) Vs Bayesian Inference (BI) | Agreement among approaches |
| --- | --- | --- | --- | --- |
| 1 | 100% | - | - | - |
| 2 | 100% | 100% | 100% | 100% |
| 3 | 100% | 100% | 100% | 100% |
| 4 | 100% | 100% | 100% | 100% |
| 5 | 90% | 100% | 100% | 97% |
| 6 | 95% | 100% | 100% | 98% |
| 7 | 95% | 95% | 100% | 97 |
| 8 | 100% | 100% | 100% | 100% |
| 9 | 90% | - | 100% | - |
| 10 | - | - | - | - |
| 11 | 90% | 100% | 100% | 97% |
| 12 | 100% | 90% | - | - |

Table S9. Chloroplasts sequences of *Oryza spp*. (<http://www.ncbi.nlm.nih.gov/genome>). Refseq, size, genes number and released date were demonstrated. Last update 15.1.2018

| **Organism Name** | **BioProject** | **Size(Mb)** | **GC%** | **Replicons** | **tRNA** | **CDS** | **Genes** | **Release Date** | **Modify Date** |
| --- | --- | --- | --- | --- | --- | --- | --- | --- | --- |
| [*Oryza alta*](https://www.ncbi.nlm.nih.gov/genome/11312?genome_assembly_id=318763) | [PRJNA387897](https://www.ncbi.nlm.nih.gov/bioproject/PRJNA387897) | 0.13518 | 39 | NC_034760.1/KF359913.1 | 37 | [87](https://www.ncbi.nlm.nih.gov/genome/browse#!/proteins/11312/318763%7COryza%20alta/chloroplast/) | 132 | 24-May-17 | 24-May-17 |
| [*Oryza australiensis*](https://www.ncbi.nlm.nih.gov/genome/10966?genome_assembly_id=205933) | [PRJNA256411](https://www.ncbi.nlm.nih.gov/bioproject/PRJNA256411) | 0.13522 | 38.95 | NC_024608.1/KJ830774.1 | 38 | [83](https://www.ncbi.nlm.nih.gov/genome/browse#!/proteins/10966/205933%7COryza%20australiensis/chloroplast/) | 129 | 29-Jul-14 | 29-Jul-14 |
| [*Oryza barthii*](https://www.ncbi.nlm.nih.gov/genome/2750?genome_assembly_id=233906) | [PRJNA289787](https://www.ncbi.nlm.nih.gov/bioproject/PRJNA289787) | 0.13467 | 38.99 | NC_027460.1/KM881634.1 | 33 | [82](https://www.ncbi.nlm.nih.gov/genome/browse#!/proteins/2750/233906%7COryza%20barthii/chloroplast/) | 123 | 14-Jul-15 | 14-Jul-15 |
| [*Oryza brachyantha*](https://www.ncbi.nlm.nih.gov/genome/10862?genome_assembly_id=282255) | [PRJNA328726](https://www.ncbi.nlm.nih.gov/bioproject/PRJNA328726) | 0.1346 | 38.98 | NC_030596.1/KT992850.1 | 38 | [83](https://www.ncbi.nlm.nih.gov/genome/browse#!/proteins/10862/282255%7COryza%20brachyantha/chloroplast/) | 129 | 12-Jul-16 | 19-Jul-17 |
| [*Oryza eichingeri*](https://www.ncbi.nlm.nih.gov/genome/54763?genome_assembly_id=318969) | [PRJNA387861](https://www.ncbi.nlm.nih.gov/bioproject/PRJNA387861) | 0.13482 | 39 | NC_034759.1/KF359912.1 | 37 | [87](https://www.ncbi.nlm.nih.gov/genome/browse#!/proteins/54763/318969%7COryza%20eichingeri/chloroplast/) | 132 | 24-May-17 | 24-May-17 |
| [*Oryza glumipatula*](https://www.ncbi.nlm.nih.gov/genome/11318?genome_assembly_id=233910) | [PRJNA289804](https://www.ncbi.nlm.nih.gov/bioproject/PRJNA289804) | 0.13458 | 38.99 | NC_027461.1/KM881640.1 | 33 | [83](https://www.ncbi.nlm.nih.gov/genome/browse#!/proteins/11318/233910%7COryza%20glumipatula/chloroplast/) | 124 | 14-Jul-15 | 14-Jul-15 |
| [*Oryza grandiglumis*](https://www.ncbi.nlm.nih.gov/genome/11320?genome_assembly_id=318764) | [PRJNA387860](https://www.ncbi.nlm.nih.gov/bioproject/PRJNA387860) | 0.13515 | 38.99 | NC_034761.1/KF359914.1 | 37 | [87](https://www.ncbi.nlm.nih.gov/genome/browse#!/proteins/11320/318764%7COryza%20grandiglumis/chloroplast/) | 132 | 24-May-17 | 24-May-17 |
| [*Oryza latifolia*](https://www.ncbi.nlm.nih.gov/genome/10967?genome_assembly_id=318759) | [PRJNA387768](https://www.ncbi.nlm.nih.gov/bioproject/PRJNA387768) | 0.13519 | 38.99 | NC_034762.1/KF359915.1 | 37 | [87](https://www.ncbi.nlm.nih.gov/genome/browse#!/proteins/10967/318759%7COryza%20latifolia/chloroplast/) | 132 | 24-May-17 | 24-May-17 |
| [*Oryza longiglumis*](https://www.ncbi.nlm.nih.gov/genome/54756?genome_assembly_id=318962) | [PRJNA387852](https://www.ncbi.nlm.nih.gov/bioproject/PRJNA387852) | 0.13564 | 38.93 | NC_034763.1/KF359918.1 | 37 | [87](https://www.ncbi.nlm.nih.gov/genome/browse#!/proteins/54756/318962%7COryza%20longiglumis/chloroplast/) | 132 | 24-May-17 | 24-May-17 |
| [*Oryza longistaminata*](https://www.ncbi.nlm.nih.gov/genome/11285?genome_assembly_id=233909) | [PRJNA289799](https://www.ncbi.nlm.nih.gov/bioproject/PRJNA289799) | 0.13457 | 38.99 | NC_027462.1/KM881641.1 | 33 | [83](https://www.ncbi.nlm.nih.gov/genome/browse#!/proteins/11285/233909%7COryza%20longistaminata/chloroplast/) | 124 | 14-Jul-15 | 14-Jul-15 |
| [*Oryza meridionalis*](https://www.ncbi.nlm.nih.gov/genome/11319?genome_assembly_id=206891) | [PRJNA86637](https://www.ncbi.nlm.nih.gov/bioproject/PRJNA86637) | 0.13456 | 39.01 | NC_016927.1/JN005831.1 | 41 | [75](https://www.ncbi.nlm.nih.gov/genome/browse#!/proteins/11319/206891%7COryza%20meridionalis/chloroplast/) | 124 | 28-Feb-12 | 28-Feb-12 |
| [*Oryza meyeriana*](https://www.ncbi.nlm.nih.gov/genome/54757?genome_assembly_id=318963) | [PRJNA387854](https://www.ncbi.nlm.nih.gov/bioproject/PRJNA387854) | 0.13613 | 38.94 | NC_034765.1/KF359921.1 | 37 | [86](https://www.ncbi.nlm.nih.gov/genome/browse#!/proteins/54757/318963%7COryza%20meyeriana/chloroplast/) | 131 | 24-May-17 | 24-May-17 |
| [*Oryza minuta*](https://www.ncbi.nlm.nih.gov/genome/10965?genome_assembly_id=277085) | [PRJNA325260](https://www.ncbi.nlm.nih.gov/bioproject/PRJNA325260) | 0.13509 | 38.96 | NC_030298.1/KU179220.1 | 39 | [89](https://www.ncbi.nlm.nih.gov/genome/browse#!/proteins/10965/277085%7COryza%20minuta/chloroplast/) | 138 | 10-Jun-16 | 10-Jun-16 |
| [*Oryza nivara SL10*](https://www.ncbi.nlm.nih.gov/genome/2841?genome_assembly_id=206845) | [PRJNA12441](https://www.ncbi.nlm.nih.gov/bioproject/PRJNA12441) | 0.13449 | 39.01 | NC_005973.1/AP006728.1 | 38 | [119](https://www.ncbi.nlm.nih.gov/genome/browse#!/proteins/2841/206845%7COryza%20nivara%20SL10/chloroplast/) | 165 | 12-Jul-04 | 11-Mar-11 |
| [*Oryza officinalis*](https://www.ncbi.nlm.nih.gov/genome/10964?genome_assembly_id=233908) | [PRJNA289798](https://www.ncbi.nlm.nih.gov/bioproject/PRJNA289798) | 0.13491 | 39 | NC_027463.1/KM881643.1 | 33 | [83](https://www.ncbi.nlm.nih.gov/genome/browse#!/proteins/10964/233908%7COryza%20officinalis/chloroplast/) | 124 | 14-Jul-15 | 14-Jul-15 |
| [*Oryza punctata*](https://www.ncbi.nlm.nih.gov/genome/10963?genome_assembly_id=241558) | [PRJNA291899](https://www.ncbi.nlm.nih.gov/bioproject/PRJNA291899) | 0.1346 | 38.97 | NC_027676.1/KM103375.1 | 41 | [100](https://www.ncbi.nlm.nih.gov/genome/browse#!/proteins/10963/241558%7COryza%20punctata/chloroplast/) | 149 | 4-Aug-15 | 4-Aug-15 |
| [*Oryza rhizomatis*](https://www.ncbi.nlm.nih.gov/genome/54789?genome_assembly_id=318995) | [PRJNA387890](https://www.ncbi.nlm.nih.gov/bioproject/PRJNA387890) | 0.1348 | 39.01 | NC_034758.1/KF359911.1 | 37 | [87](https://www.ncbi.nlm.nih.gov/genome/browse#!/proteins/54789/318995%7COryza%20rhizomatis/chloroplast/) | 132 | 24-May-17 | 24-May-17 |
| [*Oryza ridleyi*](https://www.ncbi.nlm.nih.gov/genome/10969?genome_assembly_id=318760) | [PRJNA387853](https://www.ncbi.nlm.nih.gov/bioproject/PRJNA387853) | 0.13573 | 38.92 | NC_034764.1/KF359919.1 | 37 | [87](https://www.ncbi.nlm.nih.gov/genome/browse#!/proteins/10969/318760%7COryza%20ridleyi/chloroplast/) | 132 | 24-May-17 | 24-May-17 |
| [*Oryza rufipogon*](https://www.ncbi.nlm.nih.gov/genome/457?genome_assembly_id=206777) | [PRJNA162601](https://www.ncbi.nlm.nih.gov/bioproject/PRJNA162601) | 0.13454 | 39 | NC_017835.1/JN005832.1 | 37 | [77](https://www.ncbi.nlm.nih.gov/genome/browse#!/proteins/457/206777%7COryza%20rufipogon/chloroplast/) | 122 | 9-May-12 | 9-May-12 |
| [*Oryza sativa*](https://www.ncbi.nlm.nih.gov/genome/10?genome_assembly_id=241556) | [PRJNA291900](https://www.ncbi.nlm.nih.gov/bioproject/PRJNA291900) | 0.1345 | 39 | NC_031333.1/KM103369.1 | 40 | [100](https://www.ncbi.nlm.nih.gov/genome/browse#!/proteins/10/241556%7COryza%20sativa/chloroplast/) | 148 | 5-Oct-16 | 26-Jan-17 |
| [*Oryza sativa Indica Group*](https://www.ncbi.nlm.nih.gov/genome/10?genome_assembly_id=206673) | [PRJNA17293](https://www.ncbi.nlm.nih.gov/bioproject/PRJNA17293) | 0.1345 | 39 | NC_008155.1/AY522329.1 | 0 | [64](https://www.ncbi.nlm.nih.gov/genome/browse#!/proteins/10/206673%7COryza%20sativa%20Indica%20Group/chloroplast/) | 65 | 16-Jun-06 | 15-Apr-09 |
| [*Oryza sativa Indica Group*](https://www.ncbi.nlm.nih.gov/genome/10?genome_assembly_id=303740) | [PRJNA368975](https://www.ncbi.nlm.nih.gov/bioproject/PRJNA368975) | 0.13455 | 39 | NC_027678.1/KM103382.1 | 41 | [94](https://www.ncbi.nlm.nih.gov/genome/browse#!/proteins/10/303740%7COryza%20sativa%20Indica%20Group/chloroplast/) | 143 | 4-Aug-15 | 26-Jan-17 |
| [*Oryza sativa Indica Group*](https://www.ncbi.nlm.nih.gov/genome/10?genome_assembly_id=318276) | [PRJNA318714](https://www.ncbi.nlm.nih.gov/bioproject/PRJNA318714) | 0.13455 | 39 | [Pltd: CP018170.1](https://www.ncbi.nlm.nih.gov/nuccore/CP018170.1) | 0 |  | 0 | 4-May-17 | 4-May-17 |
